# Radiomic Feature Extraction from OCT Angiography of Idiopathic Epiretinal Membranes and Correlation with Visual Acuity: A Pilot Study

**DOI:** 10.1016/j.xops.2025.100716

**Published:** 2025-01-21

**Authors:** Maria Cristina Savastano, Marica Vagni, Matteo Mario Carlà, Huong Elena Tran, Claudia Fossataro, Valentina Cestrone, Francesco Boselli, Federico Giannuzzi, Sofia Marcelli, Ilaria Biagini, Luca Boldrini, Stanislao Rizzo

**Affiliations:** 1 Ophthalmology Department, “Fondazione Policlinico Universitario A. Gemelli, IRCCS,” Rome, Italy; 2 Catholic University “Sacro Cuore,” Rome, Italy; 3 Diagnostic Imaging and Radiation Oncology, “Fondazione Policlinico Universitario A. Gemelli, IRCCS,” Rome, Italy; 4 Consiglio Nazionale delle Ricerche, Istituto di Neuroscienze, Pisa, Italy

**Keywords:** Choriocapillaris, Epiretinal membrane, OCT angiography (OCTA), Radiomics

## Abstract

**Purpose:**

To explore the correlation between radiomics features extracted from OCT angiography (OCTA) of epiretinal membranes (ERMs) and baseline best-corrected visual acuity (BCVA).

**Design:**

Retrospective observational monocentric study.

**Participants:**

Eighty-three eyes affected by idiopathic ERMs, categorized into low (≤70 letters) and high (70 letters) BCVA groups.

**Methods:**

The central 3 × 3 mm^2^ crop of structural and vascular en-face OCTA scans of superficial and deep retina slab, and choriocapillaris of each eye was selected. PyRadiomics was used to extract 86 features belonging to 2 different families: intensity-based statistical features describing the gray-level distribution, and textural features capturing the spatial arrangement of pixels. By employing a greedy strategy, 4 radiomic features were selected to build the final logistic regression model. The ability of the model to discriminate between low and high baseline BCVA was quantified in terms of area under the receiver operating characteristics curve (AUC).

**Main Outcome Measures:**

The 4 selected informative radiomic features were as follows: the difference average (glcm_DifferenceAverage), quantifying the average difference in gray-level between neighboring pixels; the informational measure of correlation (glcm_Imc1), giving information about the spatial correlation of pixel intensities inside the image; the long run low gray-level emphasis (glrlm_LongRunLowGrayLevelEmphasis), highlighting long segments of low gray-level values within the image; and the large area emphasis (glszm_LargeAreaEmphasis), which quantifies the tendency for larger zones of uniform intensity to occur.

**Results:**

No features exhibited a statistically significant difference between low and high BCVA values for the superficial and deep retinal slabs. Conversely, in the choriocapillaris layer, the glcm_DifferenceAverage and glcm_Imc1 features were significantly higher in the high BCVA group (*P* = 0.047), whereas higher values for the glrlm_LongRunLowGrayLevelEmphasis and glszm_LargeAreaEmphasis were associated with the low BCVA group (*P* = 0.047). Overall, these radiomic features predicted BCVA with an AUC (95% confidence interval) of 0.74 (0.63–0.85) and sensitivity/specificity of 0.67/0.75. During the cross-validation, the metrics remained stable.

**Conclusions:**

Radiomics features of the choriocapillaris in idiopathic ERMs showed a correlation with BCVA, with lower structural complexity and higher homogeneity, together with the presence of homogeneous areas with low-intensity pixel values, reflecting flow voids due to reduced microvascular perfusion, and were correlated with lower visual acuity.

**Financial Disclosure(s):**

The author(s) have no proprietary or commercial interest in any materials discussed in this article.

Epiretinal membrane (ERM) is the most common vitreoretinal interface disorder, with a reported prevalence of 7% to 12%.^1^ The development of idiopathic ERMs is hypothesized to result from incomplete posterior vitreous detachment and successive myofibroblastic cellular proliferation on the retinal surface. Patients with ERM may be asymptomatic or may exhibit a decrease in visual acuity and metamorphopsia, which are the main criteria for surgical excision.^2^

OCT, whose systematic use has revolutionized the evaluation of retinal diseases, plays an important role nowadays in the presurgical management of ERM as well as in the analysis of retinal changes after membrane peeling. In the earliest stage, typically asymptomatic, ERM appears as a hyperreflective band on the retinal surface. In more advanced stages, tractional effects such as retinal thickening, wrinkling of the retinal surface, and intraretinal cysts become evident.^3^

The introduction of OCT angiography (OCTA), allowing for high-quality enface visualization (“C” scan), has provided a detailed evaluation of vascular changes because of the epiretinal traction in the presurgical phase and the consequences of membrane peeling.^4^, ^5^, ^6^

Although ERM surgery has reached great standardization, it is still difficult to precisely forecast visual acuity improvement and metamorphopsia resolution, because 20% to 30% of patients do not show a satisfactory visual recovery.^7^^,^^8^ For this reason, several studies have focused attention on retinal changes in both the inner and outer layers in an attempt to define the main factor responsible for the varying degrees of visual recovery observed in different case series.^9^, ^10^, ^11^ Visual acuity and duration of the symptomatic disease have been recognized as influencing factors on the postsurgical functional outcomes. The disruption of the inner and outer segments of photoreceptors in the presurgical stage has been revealed as a crucial element of no visual gain after ERM peeling.^10^ It has also been highlighted that photoreceptor impairment is usually shown in advanced and enduring disease, which leads to chronic retinal changes.^9^

In this context, the integration of radiomics (i.e., the extraction and analysis of a large number of quantitative features from medical images)^12^ can offer a sophisticated tool to unravel intricate patterns and subtle variations within the complex structures of the eye from OCTA images.^13^^,^^14^ This approach holds significance in its ability to discover disease characteristics beyond the limits of human perception. The quantifiable features extracted with radiomics have the potential to provide clinicians with additional insights, enhancing decision-making in both diagnostic and therapeutic contexts. Furthermore, describing a medical image with its radiomic features enables the development of more stable and operator-independent predictive tools for decision support.^15^

Starting from this assumption, the aim of this pilot study was to explore the potential of radiomics in extracting OCTA features of idiopathic ERMs, correlating these findings with baseline best-corrected visual acuity (BCVA).

## Methods

### Patients and Clinical Characteristics

This is a retrospective observational, monocentric study conducted at Fondazione Policlinico Universitario A. Gemelli IRCCS, Rome, Italy, on 82 patients affected by idiopathic ERMs evaluated between January 15, 2021, and March 31, 2023. This study adhered to the tenets of the Declaration of Helsinki and all included patients provided informed consent. The research protocol was reviewed and approved by the Ethics Committee of the conducting institution (ID number 4995) and the trial was registered on clinicaltrials.gov (ID NCT05747144).

Only eyes with a documented diagnosis of symptomatic idiopathic ERM (stages 2 to 4 of Govetto et al^16^ classification) were enrolled (defined as vision loss or clinically significant metamorphopsia). Exclusion criteria included age of <18 years, any kind of secondary ERM, axial length higher than 26.5 mm, the presence of clinically significant cataract (nuclear, cortical, or posterior subcapsular), and the presence of significant concomitant ocular or systemic diseases (such as diabetic retinopathy, uveitis, severe glaucoma).

Demographic characteristics were extracted from medical records, as well as BCVA evaluation, performed by experienced optometrists and measured with Early Treatment Diabetic Retinopathy Study charts. For further analysis, patients were categorized into 2 groups based on their baseline BCVA value: low BCVA (BCVA ≤70 letters) representing the negative class, and high BCVA (BCVA >70 letters) representing the positive class.

### Scan Protocol

All images were recorded using the Solix full-range OCT (Optovue Inc), an advanced high-speed spectral-domain OCT device (version 2019 V1.0.0.317), operating at 120.000 A-scans per second with the split-spectrum amplitude-decorrelation angiography algorithm. The research participants underwent scanning procedures with the in-built AngioVue pattern, a 6.4 × 6.4 mm scan centered on the fovea. To correctly position the scanning area, a blue transverse light with predefined internal fixation was used. Poor-quality scans (due, for example, to frequent blinking or scans with significant motion artifacts) were excluded, and repeated recording of high-quality scans was performed to ensure a signal strength of ≥7/10.^17^ For each patient, structural and vascular en face scans of the in-built superficial retina slab, deep retina slab and choriocapillaris were acquired, resulting in a total of 6 scans per eye to be submitted for radiomics analysis.

### Radiomic Feature Extraction

**Figure 1:**
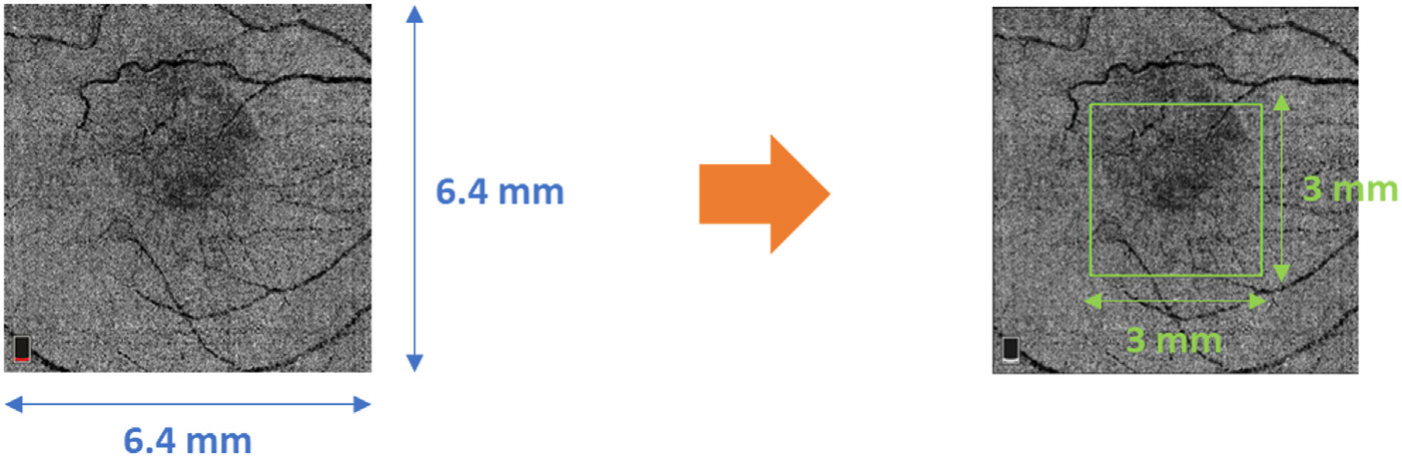
Example of the definition of a 3 × 3 mm^2^ region of interest in the central zone of an OCT angiography image at the choriocapillaris slab.

To ensure the quality of the processed images, patients with visible corrupted retinal images were excluded from the study. For each retinal image, regions of interest (ROIs) corresponding to the relevant anatomical structures were automatically delineated by considering the central region of 3 × 3 mm^2^ of the provided scans. This approach was chosen to concentrate the radiomics analysis on the most informative area of the scan and to get rid of possible noise present at the edges of the image. Figure 1 shows an example of the relevant ROI for a choriocapillaris scan.

Radiomic feature extraction was performed by using PyRadiomics*,* a Python package designed for the extraction of quantitative features from an ROI within medical images.^18^ These features included first-order statistics computed from the pixel intensities histogram (e.g., mean, variance) summarizing the pixel intensity distributions, and textural features that provide information on the local spatial distribution of the gray levels within the ROI. The textural features are computed based on the following matrixes: (1) gray-level cooccurrence matrix (GLCM), which measures the frequency of pairs of pixel intensities occurring in specified spatial relationships, providing insights into the contrast and spatial structure of the tissue; (2) gray-level run length matrix (GLRLM), which captures the length of consecutive pixels with the same intensity; (3) gray-level size zone matrix (GLSZM), which measures the size of homogeneous regions of pixels; and (4) gray-level dependence matrix, which quantifies how much neighboring pixels depend on each other in terms of intensity. Shape features were not computed because the ROI was defined as a rectangular shape.

### Statistical Analysis and Model Building

For each imaging modality separately, statistical analysis was performed to keep only features that can significantly discriminate between low and high baseline BCVA. First, a univariate analysis using the Wilcoxon–Mann–Whitney test was conducted to identify features that exhibited statistically significant differences between the 2 classes (i.e., low-high baseline BCVA). Subsequently, the *P* values were adjusted for multiple testing using the Benjamini–Hochberg procedure. The significance level was set at 0.05. To mitigate multicollinearity among the features, the Pearson cross-correlation coefficient was then computed: features exhibiting a high correlation (above the threshold of 0.9) were excluded.

To ensure the consistency of radiomic features’ ranges across patients, feature values were normalized with the z-score before further analysis.

In the final step, a logistic regression model was implemented utilizing a greedy approach as feature selection, where all possible combinations of the retained features were tested for model building. The generalization capability of the final model was assessed using a 3-fold cross-validation repeated 5 times in the internal data set.

The ability of the model to discriminate between low and high baseline BCVA was quantified in terms of area under the receiver operating characteristics (ROC) curve (AUC). Accuracy, sensitivity, and specificity when using the Youden cut-off (the cut-off maximizing correct classifications)^19^ were computed. The 95% confidence intervals (CI) were also provided: the 95% CI was computed through bootstrapping for the AUC while using normal approximation for the accuracy, sensitivity, and specificity.

Statistical analysis, model building, and evaluation were performed in Python 3.7. Descriptive data were presented as median (interquartile range).

### Radiomic Model Calibration

Assessing the calibration of a binary prediction model is crucial to evaluate its reliability and predictive performance. The internal calibration curve, which gives information on the agreement between the radiomic model’s predicted probabilities and the actual outcomes, was first computed to measure the model’s ability to provide accurate probability estimates. Subsequently, the calibration performance was also assessed by analyzing the shape and distribution of the calibration belt plot,^20^ which provides the 95% confidence band of the calibration curve, allowing for a comprehensive evaluation of model calibration. Deviations from the ideal calibration line (a 45-degree diagonal line) indicate potential issues with model calibration. Finally, a likelihood-based statistical test based on a polynomial logistic regression was performed to compare the probabilities predicted by the model with the observed outcomes,^20^ with a significance level of 0.05.

### Radiomic Model as a Decision Support Tool

Analyzing the results from the posttest probability given the pretest probability and likelihood ratios (LR) permits the assessment of the utility of the proposed radiomic model as a decision support tool. Indeed, a useful model should transform the pretest probability into a posttest probability which can effectively support clinicians in their decisions, providing reliable predictions.

The pretest (or prior) probability represents the initial likelihood of belonging to the positive class, namely having a high BCVA (BCVA >70 letters), for a patient before the application of the model. In this study, the pretest probability is given by the prevalence (i.e., the proportion of the considered patient cohort having high BCVA). On the other hand, the posttest probability is the updated probability of the BCVA value being high after the application of the model. It reflects indeed the combined influence of the pretest probability and the model’s diagnostic performance. The positive (LR+) and negative (LR–) LRs represent how many times more (or less) likely patients with the considered condition (i.e., having high BCVA value) are to have a positive or negative model prediction, respectively, when compared with a patient without the considered condition (i.e., having low BCVA value). The more the LR deviates from 1, the stronger the evidence for the presence or absence of the considered condition.^21^

Therefore, the posttest probability tells how much more (or less) certain we can be about the condition's presence or absence after we have applied the radiomic model. It helps guide decision-making in clinical practice based on the additional information provided by the model.

For this purpose, we used an online diagnostic calculator tool (http://araw.mede.uic.edu/cgi-bin/testcalc.pl?DT=0&Dt=0&dT=0&dt=0&2).

## Results

Out of 82 enrolled patients, 3 of them presented corrupted images (Fig S2, available at www.ophthalmologyscience.org, shows an example of low-quality images for an excluded patient): the final data set therefore comprised 83 eyes from 79 patients with the following baseline visual distribution: low BCVA −44 eyes versus high BCVA −39 eyes. The mean age was 69 ± 6 years (range 58–79). Overall, 28 eyes had a stage 2, 39 eyes a stage 3, and 16 eyes a stage 4 ERM.

### Radiomic Feature Extraction and Radiomic Model Development

A total of 86 radiomic features were initially extracted with PyRadiomics for each imaging modality.

After conducting univariate analysis and adjusting *P* values by using the Benjamini and Hochberg procedure to eliminate false positive findings, no features exhibited a statistically significant difference between low and high BCVA values for the superficial and deep scans in both OCTA and en face. However, in the case of the OCTA choriocapillaris scans, 41 features remained statistically significant. The computation of the Pearson correlation coefficients further refined the features’ selection, leaving 5 features that exhibited noncorrelated relationships. The subsequent analyses will therefore describe results for the sole choriocapillaris characterization.

By employing a greedy strategy, the following 4 radiomic features were selected to build the final logistic regression model:

1. The difference average from the GLCM (glcm_DifferenceAverage) was selected which quantifies the average difference in gray-level values between neighboring pixels based on their spatial relationships. This feature is thus a measure of contrast, with higher values indicating greater contrast or heterogeneity in the image. In other words, a texture with a higher different average may appear visually noisier.
2. The informational measure of correlation from the GLCM (glcm_Imc1), was selected which provides information about the spatial correlation of pixel intensities within the image. Although this feature is more difficult to be directly linked to a visual characteristic of the image, it helps assess the complexity of tissue texture. In particular, high correlation values indicate a strong linear relationship between the gray levels, whereas lower values suggest an image texture with more rapid changes and variations in pixel intensity. It is worth noticing that complexity differs from heterogeneity: a texture can be nonuniform yet exhibit low complexity because the lack of uniformity might follow a structured pattern that is not immediately apparent to the human eye.
3. The long run low gray-level emphasis from the GLRLM (glrlm_LongRunLowGrayLevelEmphasis), was selected which highlights the presence of long segments of low gray-level values within the image.
4. The large area emphasis from the GLSZM (glszm_LargeAreaEmphasis) was selected which indicates the presence of larger homogeneous regions within the image.

**Figure 3:**
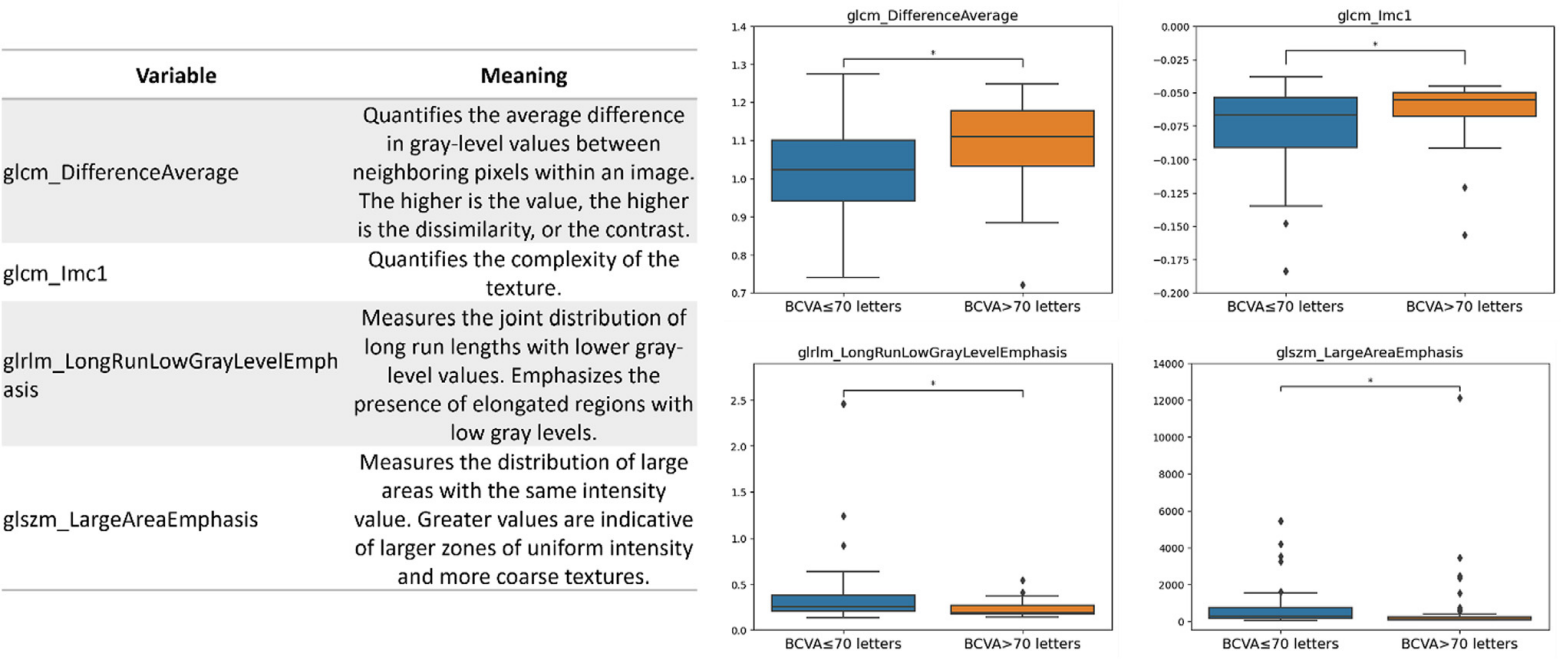
On the right, boxplots show the distribution of the selected radiomic features used as covariates for model building in the 2 groups. On the left, a table summarizes the image's textural characteristics reflected by each of the selected features. BCVA = best-corrected visual acuity; GLCM = gray-level cooccurrence matrix.

Figure 3 shows the boxplots of the aforementioned radiomic features used in the final predictive model for the 2 classes, along with the textural image characteristic they reflect. The *P* value obtained with the Wilcoxon text and corrected by the Benjamin procedure for differentiating between the 2 classes was equal to 0.047 for all the selected features.

**Figure 4:**
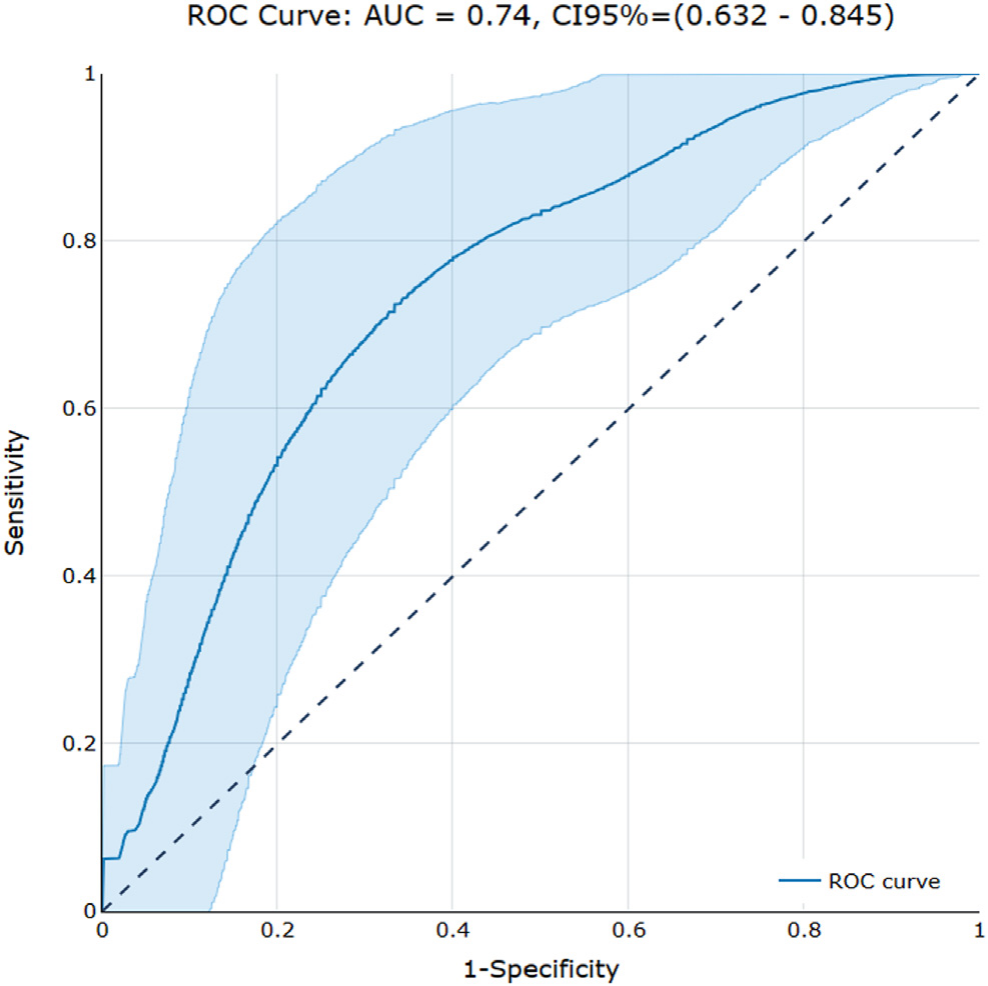
Receiver operating characteristics (ROC) curve for the proposed radiomic model. The diagonal line represents the performance expected by random chance. The light blue band represents the 95% confidence interval (CI). AUC = area under the ROC curve.

Figure 4 illustrates the ROC curve for the proposed predictive model, whereas Table 1 summarizes the performance of the developed model obtained from both model fitting and internal 3-fold cross-validation. The predictive performance obtained from the model fitting is as follows: AUC = 0.74 (95% CI, 0.63–0.85), accuracy = 0.71 (95% CI, 0.61–0.81), sensitivity = 0.67 (95% CI, 0.52–0.81), specificity = 0.75 (95% CI, 0.62–0.88). During the crossvalidation, the metrics remained stable with a slight decrease, which is expected due to the inherent variability introduced by the technique.

**Table 1:** Performance of the Proposed Radiomic Model

	AUC	Accuracy	Sensitivity	Specificity
Model fitting	0.74 (0.63–0.85)	0.71 (0.61–0.81)	0.67 (0.52–0.81)	0.75 (0.62–0.88)
Cross-validation	0.68 (0.12)	0.67 (0.07)	0.66 (0.23)	0.67 (0.17)

The 95% confidence intervals are reported for model fitting metrics in parentheses. Mean and standard deviation (in parentheses) are reported for the cross-validation.

AUC = area under the receiver operating characteristics curve.

To assess the potential impact of age on choriocapillaris, we incorporated it as a covariate in the logistic regression model alongside the selected radiomic features. The ROC curves for the model with and without age were overlapping (Fig S5, available at www.ophthalmologyscience.org), confirming that the inclusion of age does not significantly impact the model's performance. Moreover, no statistically significant difference between the AUC of the models with and without age as a covariate was reported using the DeLong method (*P* = 0.67).

### Calibration of Radiomic Model

**Figure 6:**
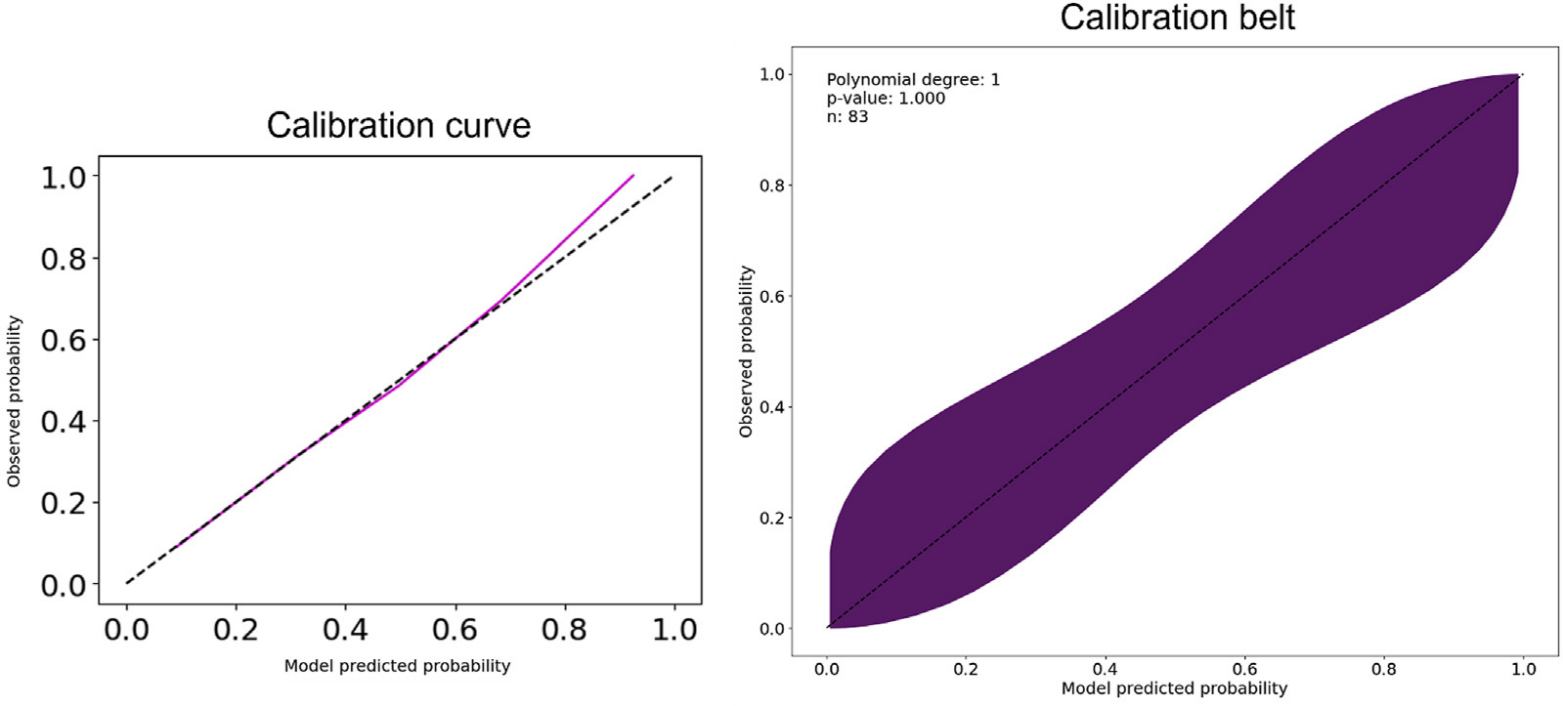
Left side: calibration plot for the proposed radiomics model (magenta). The black dashed line indicates the ideal calibration. Right side: calibration belt plot displaying the observed frequencies against the predicted probabilities. The calibration belt (purple area) provides the 95% confidence interval for the predicted probabilities. The dashed black line indicates ideal calibration.

Figure 6 illustrates the calibration curve (left side) and the calibration belt (right side), displaying the observed probabilities against the model-predicted probabilities. The calibration curve lays along the bisector for most of the observed probability range, whereas the calibration belt providing the 95% CI (purple area) contains the ideal calibration line. The results indicated that the proposed radiomic model was well-calibrated across the entire probability spectrum. Moreover, because the *P* value obtained from the calibration belt was high (*P* = 1), we failed to reject the null hypothesis that the expected and observed probabilities were equal.

### Assessment of Radiomic Model as a Decision Support Tool

Table 2 summarizes the results obtained from the likelihood analysis.

**Table 2:** Pretest Probability, Likelihood Ratios, and Posttest Probability for Positive and Negative Model Prediction

	Positive Model Prediction (Predicted Class 1)	Negative Model Prediction (Predicted Class 0)
Pretest probability	47%	47%
Likelihood ratio (95% CI)	LR+ = 2.67 (1.53−4.66)	LR− = 0.44 (0.28−0.72)
Posttest probability (95% CI)	70% (58%−81%)	28% (20%−39%)

CI = confidence interval; LR– = likelihood negative ratio; LR+ = likelihood positive ratio.

The prior test probability of the condition was equal to 47%. Considering that the positive and the negative classes were given by patients with BCVA >70 letters and patients with BCVA ≤70 letters, respectively, the LR+ of 2.67 indicates that the likelihood of obtaining a positive prediction for a patient with BCVA >70 letters by applying the radiomic model is 2.67 times higher than for a patient with BCVA ≤70 letters. On the other hand, an LR− of 0.44 indicates that it is 0.44 times less probable to have a negative prediction when a patient presents BCVA >70 letters compared with a patient presenting BCVA ≤70 letters.

As a consequence, the posttest probability for a positive and negative model prediction was equal to 70% and 28%, respectively. These changes in posttest probabilities show the impact of the radiomic model on the assessment of the condition's likelihood. The radiomic model provides valuable information, particularly in increasing confidence when the model prediction is positive.

**Figure 7:**
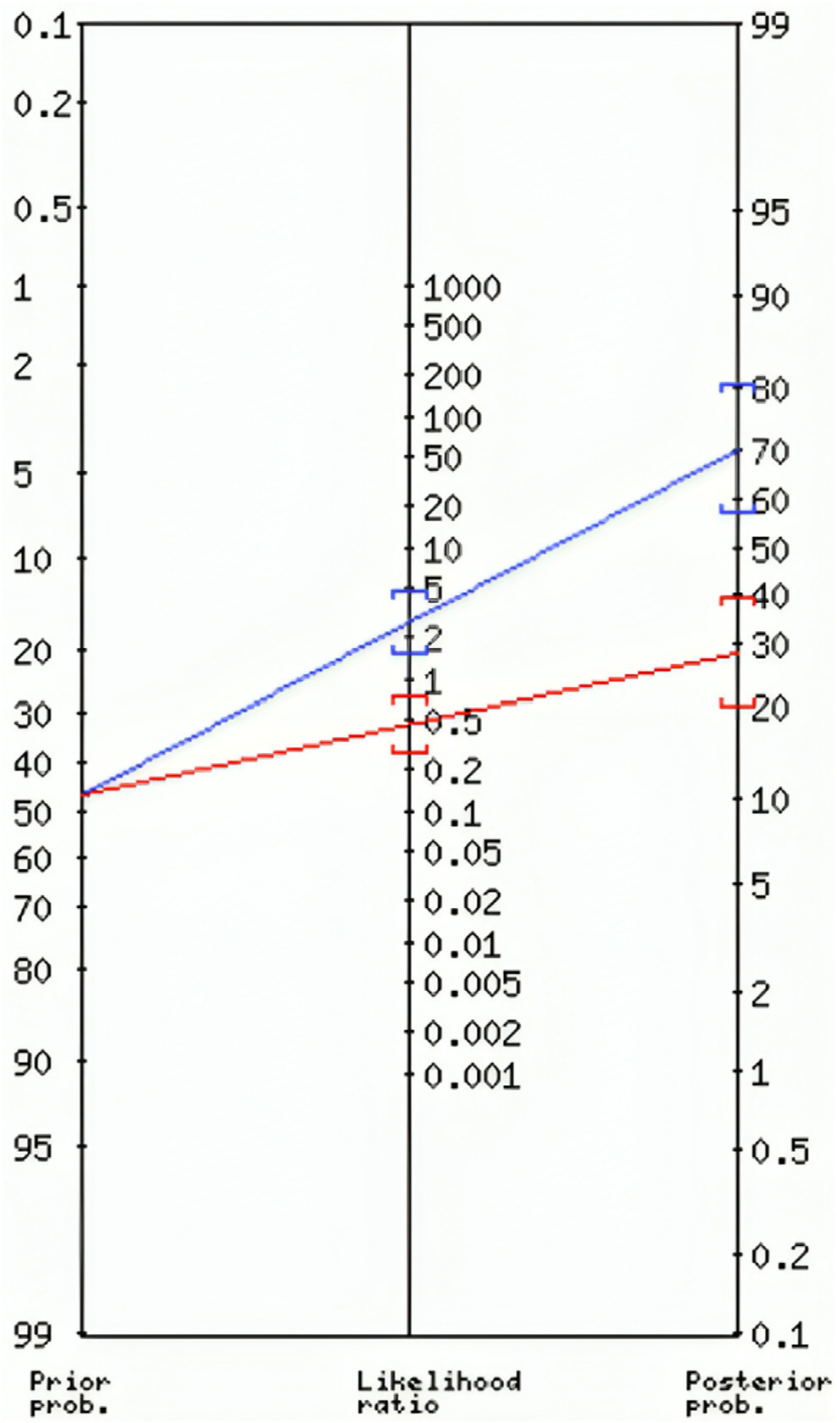
Fagan's nomogram illustrating the estimate of the probability of a patient having a high best-corrected visual acuity value based on the model’s prediction. Two lines are drawn from the pretest probability to the likelihood ratio points corresponding to the radiomic model’s performance. The intersection of the drawn lines with the posttest probability scale gives an estimate of the posttest probability of the patient having a high value of best-corrected visual acuity. Figure obtained from an online diagnostic test calculator tool (http://araw.mede.uic.edu/cgi-bin/testcalc.pl?DT=&Dt=&dT=&dt=&2).

Figure 7 shows the Fagan nomogram which enables a graphical derivation of the posttest probability for a positive/negative model prediction based on the pretest probability and the positive/negative LRs (LR+ and LR–) obtained from the model's performance metrics.^22^

## Discussion

Our study focused on the radiomic characterization of OCTA scans in idiopathic ERMs and their correlation with BCVA. Despite utilizing various imaging modalities, including superficial capillary plexus (SCP), deep capillary plexus (DCP), and choriocapillaris imaging, in either OCTA and structural enface, only the scans focused on the choriocapillaris layer exhibited statistically different features between eyes with low (≤70 letters) and high (>70 letters) BCVA.

In particular, 4 textural radiomic features emerged as significant in distinguishing these groups (Fig S2, available at www.ophthalmologyscience.org). The difference average and the informational measure of correlation, both from the GLCM, reflect different aspects of image texture complexity. Higher values for these metrics in the high BCVA group suggest a more intricate and structured pattern in the choriocapillaris, potentially reflecting healthier or more intact vascular branching. In contrast, lower values in the low BCVA group may indicate a loss of structural complexity, possibly due to choriocapillaris rearrangement.

Conversely, long run low gray-level emphasis (GLRLM) and large area emphasis (GLSZM) were associated with low BCVA. These features reflect the presence of elongated low-intensity regions and larger homogeneous areas, respectively, both of which may suggest areas of flow void or disrupted vascular structure in the choriocapillaris.

These findings suggest that lower structural complexity and higher homogeneity in the choriocapillaris, along with the presence of homogeneous areas of low-intensity pixel values, reflect pathologic changes such as flow voids or capillary loss, and are associated with poorer visual outcomes. In contrast, greater complexity and heterogeneity correlate with better BCVA, likely indicating a healthier microvascular network capable of sustaining nutrient delivery to the retina (Fig S8, available at www.ophthalmologyscience.org).

By leveraging the 4 aforementioned radiomic features, we developed a radiomics-based logistic regression model to objectively characterize the patient’s BCVA from choriocapillaris OCTA vascular layers in ERM disease.

The predictive performance of the proposed model demonstrated a good balance between specificity and sensitivity, as shown by the ROC curve (Fig 3), with well-calibrated performance across the entire probability spectrum (Fig 4). The performance obtained from the fitting of the model remained stable during the cross-validation test, indicating good generalizability of our findings.

Previous research has shown various findings regarding the correlations between retinal vascular characteristics measured using OCTA and visual acuity in ERMs.^23^ Lower vascular density in the DCP and SCP was observed to be connected with decreased visual acuity in eyes with ERM by Bacherini et al.^5^ Conversely, Yuce et al^24^ reported that in eyes with ERM, there was a negative correlation between lower visual acuity and larger vessel density in the DCP. Conversely, according to other research, there is no connection between the vessel density in the SCP or DCP and visual acuity in eyes with ERM.^25^ The disparity in image processing techniques and the combined examination of capillaries and big veins may be the cause of this discrepancy. Moreover, extracted features of OCTA scans analyzed with image processing tools have been demonstrated as indicators of ERM severity and visual impairment: increased average vessel diameter, decreased skeleton density, and decreased vessel tortuosity are all associated with inner retinal folding and thickened inner nuclear layer, indicating more severe ERMs with poorer BCVA.^26^ Finally, Cheng et al^27^ recently demonstrated the significance of isolating the capillaries and big arteries for the examination of OCTA parameters in ERM due to their entirely distinct associations with visual acuity. Greater vascular tortuosity, greater fractal dimension, and higher vessel density of the retinal capillaries were all associated with improved vision. Conversely, worsening retinal thickness and folding in more severe ERMs would lead to a less distinct retinal capillary network in OCTA.^27^ The lack of distinction between capillaries and large vessels could justify the absence of correlation between the radiomic features of the SCP/DCP with BCVA that we highlighted in this study. Conversely, the choriocapillaris, being composed only of small vessels, could be less impacted by these limitations and be more suitable for radiomic analysis.

Of note, the exploration of choroid and choriocapillaris has recently gained significant attention due to the advancements in technology. The analysis of choroidal vascularity index changes after vitreomacular surgery was recently reported by Rizzo et al,^28^ who demonstrated a reduction of choroidal vascularity index after ERM removal suggesting structural and functional changes affecting the choroidal layer after surgery. As demonstrated in several reports, choroid and choriocapillaris are dynamic tissues that might go through extensive remodeling in ocular and systemic pathologic conditions or after medical and surgical treatment.^17^^,^^29^, ^30^, ^31^, ^32^

Although our ultimate goal was to predict postsurgery BCVA at 6 months using diagnostic images, the limitations of our data set necessitated an exploratory approach. Consequently, our primary focus shifted to assessing the feasibility of characterizing baseline BCVA at the time of diagnosis. Successfully establishing this connection will pave the way for future efforts to predict postsurgery visual outcomes with greater precision and reliability. Apart from that, our study had several shortfalls, primarily due to its retrospective nature and the small cohort of patients. In addition, the lack of a control group and the absence of a subdivision of radiomic characteristics based on the ERM stage are clear limitations to be addressed in further research. Finally, correlating specific radiomic features, very rigidly defined, with the more subjective BCVA evaluation, may have led to a possible accuracy mismatch, highlighting the need for further research to validate our findings.

To the best of our knowledge, there are no other studies in literature analyzing OCT scans to predict BCVA using machine learning models utilizing radiomic features as covariates for ERM. However, it is mandatory to mention the shortfalls of our research. First, the model has been developed using a monocentric, retrospective, relatively small patient cohort, which has intrinsic limitations. Second, because this was a pilot study, we focused on preoperative features only, although further research may highlight whether the evolution of radiomics characteristics could demonstrate a predictive value on visual outcomes. Furthermore, radiomic features are based on pixel-by-pixel interactions, and the correlation with comprehensive OCT scans or functional data may not be as direct as reported. Future studies, performed in longitudinal, multicentric, and multidevice subsets are necessary to validate our model and to confirm the reported data.

In conclusion, the application of radiomics in OCT/OCTA imaging analysis allowed us to extract ultrastructural radiomic features, unidentifiable to the human eye, suggesting that the architecture of the choriocapillaris in ERM has a significant impact on preoperative BCVA. The findings of this pilot study can shed light on the role of the choriocapillaris as a prognostic factor before ERM surgery, in the meantime being a useful tool for predictive functional results. Nevertheless, more studies on this topic with large sample sizes, different devices, and different conditions are necessary to investigate how the application of radiomics may impact clinical practice in ophthalmology.
