# Supplementary figures and images for: Radiomic Feature Extraction from OCT Angiography of Idiopathic Epiretinal Membranes and Correlation with Visual Acuity: A Pilot Study
Source: Ophthalmol Sci. 2025 Jan 21;5(3):100716. doi: 10.1016/j.xops.2025.100716 (PMC11919415; doi:10.1016/j.xops.2025.100716)

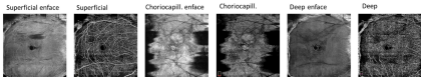

Supplementary Figure 2: Example of excluded patient presenting corrupted images.

Supplement: Figure S2 [file mmc1.pdf]
