# Supplementary material for: Radiomic Feature Extraction from OCT Angiography of Idiopathic Epiretinal Membranes and Correlation with Visual Acuity: A Pilot Study
Source: Ophthalmol Sci. 2025 Jan 21;5(3):100716. doi: 10.1016/j.xops.2025.100716 (PMC11919415; doi:10.1016/j.xops.2025.100716)
Supplement: Figure S5 [file mmc2.pdf]

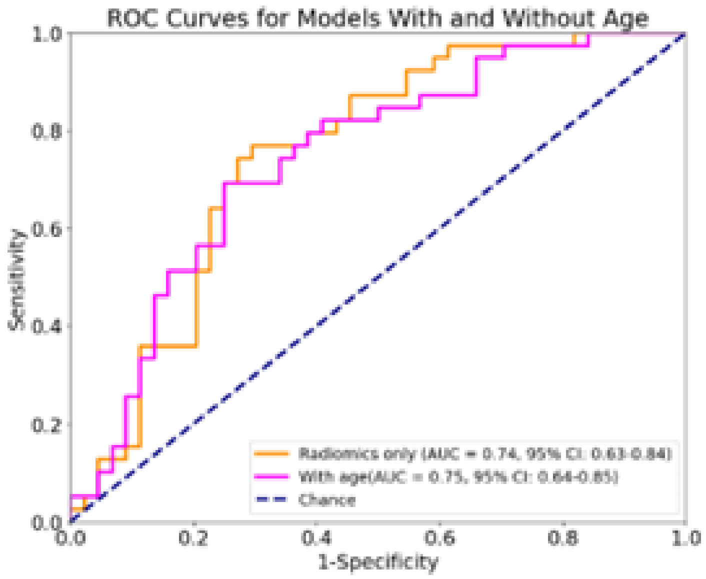

Supplemental Figure 5. ROC curves for the model with and without age were overlapping, confirming that the inclusion of age does not significantly impact the model's performance. Moreover, the statistical significance of the difference in AUC between the two models was tested using the DeLong method, resulting in a p-value of 0.67. This result indicates no statically significant difference between the AUC of the models with and without age as a covariate.
