# Supplementary material for: Radiomic Feature Extraction from OCT Angiography of Idiopathic Epiretinal Membranes and Correlation with Visual Acuity: A Pilot Study
Source: Ophthalmol Sci. 2025 Jan 21;5(3):100716. doi: 10.1016/j.xops.2025.100716 (PMC11919415; doi:10.1016/j.xops.2025.100716)
Supplement: Supplementary Figure 8 [file mmc3.pdf]

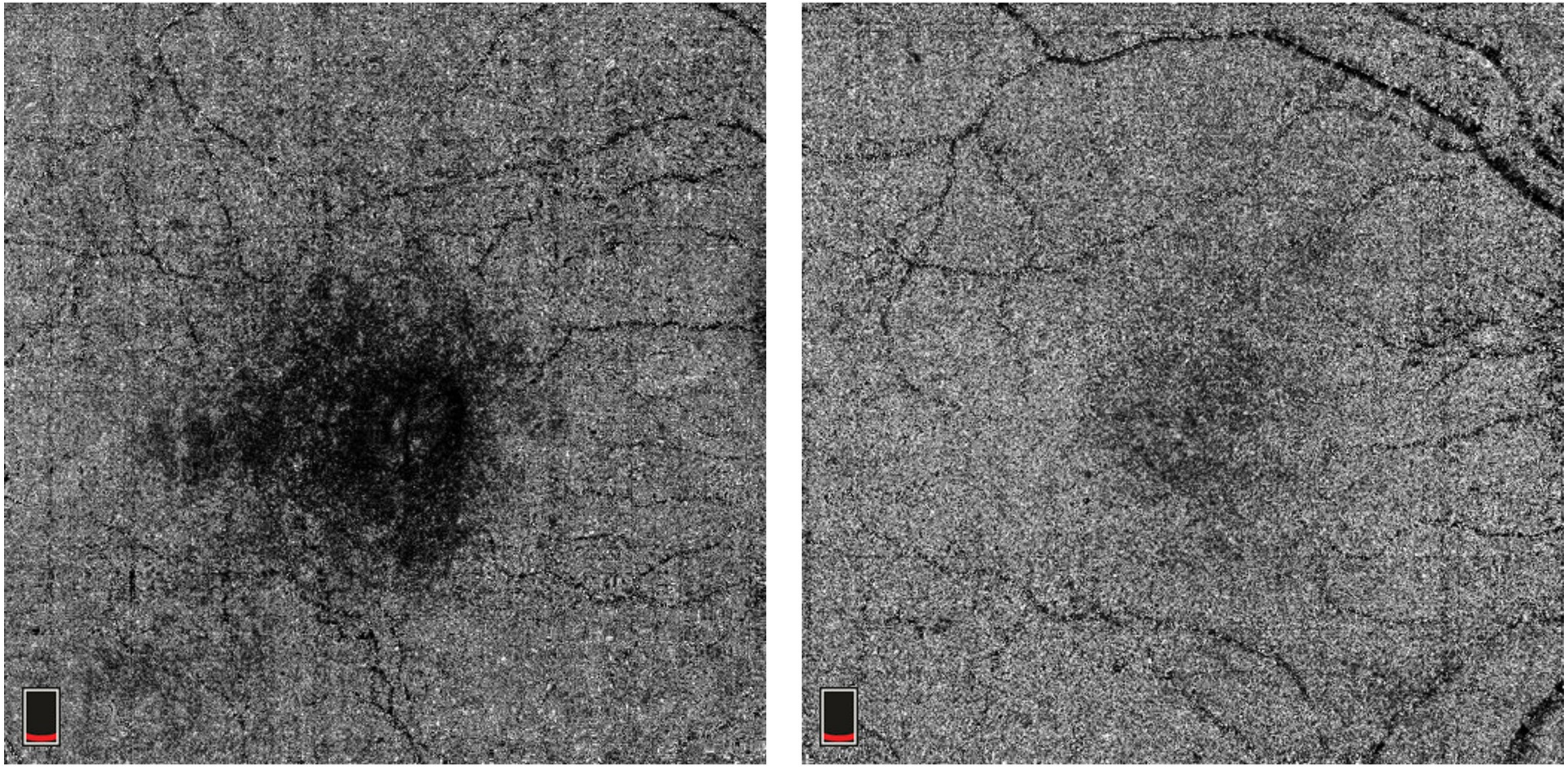

Patients with poor visual acuity (low BCVA, Figure left) exhibited central areas of diffuse low-gray-level intensity, representing widespread flow voids or microvascular insufficiency. In contrast, patients with good VA (high BCVA, Figure right) have more heterogeneous and complex microvascular patterns, reflecting healthier choriocapillaris structures and better nutrient delivery.
